# Notes and Notices

**Published:** 1889-03

**Authors:** 


### NOTES AND NOTICES.

REMOVAL.—Dr. H. S. Phillips, from Canonsburg, to 328 Fifth Avenue, Pittsburg, where he will be associated with Dr. W. D. King.

THE CLINICAL RECORD is a new journal started by W. A. Chatterton, of Chicago. It is to give brief extracts of clinical data; volume began in January; price, one dollar per year.

THE ANNALS OF SURGERY began its ninth volume with its January issue. This is perhaps the only English journal devoted exclusively to surgery; it has an American and an English editor; it gathers its data from the surgical reports of the world. To those who devote time and attention to surgery, the *Annals* is a necessity. It is published by Messrs. J. H. Chambers & Co., 914 Locust Street, St. Louis.

BROOKLYN SUBSCRIBER.—A subscriber to this journal recently mailed (Feb. 20th) us from Brooklyn an *unsigned* subscription blank, with a two-dollar note inclosed. We shall be glad to have the name and address sent us.

MORPHIA *vs.* HOMŒOPATHY.—But, says the morphinist, when my patient is suffering, he wants immediate relief, and the hypodermic does it without wading through the intricacies of an overloaded materia medica. Granted—but do not claim to be a follower of Hahnemann's teaching. Low potencies and palliatives go hand in hand *versus* high potencies and strict application of the law of similarity. We do not infringe on your liberty, but please do not let it run to weeds in the form of license. Charity to all, but do not abuse those who pride themselves upon being strict followers of the father of Homœopathy.—S. L. in *Med. Institute.*

THE SCIENTIFIC AMERICAN, published by Munn & Co., New York, during more than forty years, is, beyond all question, the leading paper relating to science, mechanics, and inventions published on this continent. Each weekly issue presents the latest scientific topics in an interesting and reliable manner, accompanied with engravings prepared expressly to demonstrate the subjects. The *Scientific American* is invaluable to every person desirous to keep pace with the inventions and discoveries of the day.

FRENCH LIQUERS.—Absinthe, the favorite intoxicant of the French, is almost always manufactured with alcohols of industry, ill rectified, rendered green by the addition of sulphate of copper, and saturated with resin, so as to give it the beautiful greenish-white precipitate produced by pouring water on it, and which drinkers so much admire. "Vermouth," another favorite liquor, is adulterated with hydrochloric or sulphuric acid in order to give it a pungent taste. "Kirsch" is extracted from the leaves of the cherry-laurel. Rum is manufactured always with alcohol distilled from beet-root, to which is added ether and formic acid. The "bouquets" of brandies are manufactured by the action of sulphuric acid on castor oil. The coloring matters employed are extracted from logwood, the elder, sorrel, fuchsine, and coal. Such is the poison which is daily consumed by the Parisians.

